# Impact of a standardized perioperative care protocol on functional and radiographic outcomes following transforaminal lumbar interbody fusion for degenerative spondylolisthesis: a 2-year randomized controlled trial

**DOI:** 10.3389/fsurg.2025.1679851

**Published:** 2025-12-18

**Authors:** Yixin Zhao, Jiangnan Wu, Zhenzhen Zhang, Yuqian Wang, Baoli Li

**Affiliations:** Department of Spine Surgery, The Third Hospital of Hebei Medical University, Shijiazhuang, China

**Keywords:** standardized perioperative care protocol, Enhanced Recovery After Surgery (ERAS), lumbar spondylolisthesis, spinal fusion, transforaminal lumbar interbody fusion, randomized controlled trial, rehabilitation

## Abstract

**Objective:**

To evaluate the efficacy of a comprehensive, standardized perioperative care protocol (SPCP) vs. conventional care on functional recovery, radiographic outcomes, and quality of life in patients undergoing transforaminal lumbar interbody fusion (TLIF) for low-grade degenerative lumbar spondylolisthesis.

**Methods:**

This was a single-center, prospective, randomized controlled trial conducted between January 2018 and June 2023. A total of 382 patients were randomized to either the SPCP group (*n* = 191) or the conventional care (control) group (*n* = 191). The SPCP incorporated preoperative education, nutritional optimization, standardized anesthesia and surgical techniques, and a structured, goal-directed postoperative rehabilitation program. The control group received routine institutional care. The primary outcome was the change in the Oswestry Disability Index (ODI) score at 2-year follow-up. Secondary outcomes included Japanese Orthopaedic Association (JOA) scores, Visual Analog Scale (VAS) for back and leg pain, Short Form-36 (SF-36) quality of life scores, radiographic outcomes (fusion rate, segmental lordosis, disc height), length of hospital stay (LOS), and postoperative complications. Assessments were performed at baseline, 3 months, 6 months, 1 year, and 2 years.

**Results:**

At the 2-year follow-up, the SPCP group demonstrated a significantly greater improvement in ODI scores compared to the control group (mean change: −30.0 vs. −25.5 points; mean difference: −4.5, 95% CI: −5.9 to −3.1; *P* < 0.001). The SPCP group also showed superior JOA scores (27.5 vs. 23.1; *P* < 0.001), lower VAS back pain scores (1.1 vs. 2.4; *P* < 0.001), and higher SF-36 Physical Component Summary (PCS) scores (48.2 vs. 42.5; *P* < 0.001). Radiographically, the SPCP group achieved a higher fusion rate at 2 years (94.4% vs. 88.7%; *P* = 0.018) and better maintenance of segmental lordosis. Mean LOS was significantly shorter in the SPCP group (7.5 ± 2.1 vs. 9.8 ± 2.5 days; *P* < 0.001), with a lower overall 90-day complication rate (8.4% vs. 19.4%; *P* = 0.002).

**Conclusion:**

Implementation of a comprehensive SPCP significantly enhances long-term functional recovery, improves radiographic fusion rates, elevates quality of life, and reduces complications and hospital stay for patients undergoing TLIF for degenerative spondylolisthesis. This protocol-driven approach represents a valuable strategy for optimizing patient outcomes and healthcare efficiency in spine surgery.

**Clinical trial registration:**

ClinicalTrials.gov, identifier NCT07104448.

## Introduction

Degenerative lumbar spondylolisthesis, a common pathology characterized by the forward slippage of a vertebra, affects up to 11.5% of the elderly population and is a primary contributor to lumbar spinal stenosis, resulting in chronic low back pain, radiculopathy, and diminished quality of life (1, 2). When conservative treatments fail, surgical intervention is often warranted. Transforaminal lumbar interbody fusion (TLIF) has emerged as a widely accepted procedure, offering robust biomechanical stability, direct neural decompression, and restoration of sagittal alignment through a single posterior approach (3).

Despite the technical success of TLIF, patient outcomes can be highly variable. Postoperative recovery is influenced not only by the surgical procedure itself but by a multitude of perioperative factors, including patient comorbidities, pain management strategies, and rehabilitation protocols (4). Traditional care pathways are often fragmented, leading to inconsistencies in practice, prolonged hospital stays, and suboptimal functional recovery (5). Enhanced Recovery After Surgery (ERAS) protocols have demonstrated significant benefits across various surgical disciplines by standardizing care to mitigate the surgical stress response and accelerate recovery (6). However, the application of comprehensive, standardized protocols in spine surgery, particularly for complex procedures like TLIF, is still evolving and often lacks rigorous, long-term evidence from randomized controlled trials (5, 7).

Existing protocols frequently focus on isolated elements of perioperative care, such as pain control or early mobilization, without integrating them into a holistic, evidence-based pathway that spans the entire patient journey—from preoperative optimization to long-term follow-up (8). We hypothesized that a multifaceted Standardized Perioperative Care Protocol (SPCP), which combines preoperative patient conditioning, intraoperative best practices, and a structured, goal-oriented postoperative rehabilitation plan, would lead to superior functional and radiographic outcomes compared to conventional, non-protocolized care. This study aims to rigorously evaluate the impact of such a protocol on patients undergoing TLIF for low-grade degenerative spondylolisthesis over a 2-year follow-up period.

## Materials and methods

### Study design and participants

This study was a single-center, prospective, parallel-group randomized controlled trial, approved by the Institutional Review Board of The Third Hospital of Hebei Medical University. The study was conducted in accordance with the principles of the Declaration of Helsinki (9) and the CONSORT 2010 statement (10). All participants provided written informed consent prior to enrollment. This study was retrospectively registered with ClinicalTrials.gov (Identifier: NCT07104448).

From January 2018 to June 2023, we assessed 850 patients with symptomatic, single-level (L3-L4, L4-L5, or L5-S1) low-grade (Meyerding Grade I or II) degenerative spondylolisthesis who had failed at least 6 months of conservative therapy. Inclusion criteria included age between 40 and 75 years and suitability for TLIF surgery. Exclusion criteria were high-grade spondylolisthesis (>Grade II), previous lumbar surgery, spinal infection, tumor, trauma, severe osteoporosis (T-score < −3.0), or significant medical comorbidities (ASA physical status > III) precluding major surgery.

### Randomization and blinding

Eligible patients were randomly allocated in a 1:1 ratio to either the SPCP group or the control group. Randomization was performed using a computer-generated block randomization sequence (blocks of 4 and 6) created by a statistician not involved in patient recruitment or care. Allocation was concealed using sequentially numbered, sealed, opaque envelopes. While participants and surgeons could not be blinded to the treatment allocation due to the nature of the intervention, the outcome assessors, radiographic evaluators, and data analysts were blinded throughout the study.

### Surgical procedure

All patients underwent a single-level open TLIF performed by one of three senior spine surgeons with over 15 years of experience. A standard midline posterior approach was used, followed by bilateral pedicle screw placement (DePuy Synthes Expedium® system), unilateral facetectomy for access to the disc space, discectomy, endplate preparation, and insertion of a PEEK interbody cage (Medtronic Capstone®) filled with autologous bone graft. Decompression of the neural elements was performed as required.

### Interventions

#### Standardized perioperative care protocol (SPCP) group

Patients received a multi-modal, standardized protocol:

• Preoperative Phase: Comprehensive education on the surgical process and recovery expectations, nutritional screening and supplementation if needed, and instruction in deep breathing exercises and basic bed mobility.
• Intraoperative Phase: Standardized anesthetic regimen (avoiding long-acting opioids), goal-directed fluid therapy, and maintenance of normothermia.
• Postoperative Phase: Multimodal, opioid-sparing analgesia (scheduled paracetamol and NSAIDs, with opioids for breakthrough pain only). Urinary catheter removal on postoperative day (POD) 1. Structured mobilization guided by a physiotherapist: sitting out of bed on POD 1, ambulating with a walker on POD 2, stair climbing practice on POD 3. Discharge was planned when patients met specific functional criteria (e.g., independent ambulation over 30 meters, adequate pain control on oral analgesia).

#### Control group (conventional care)

Patients received standard institutional care, which was not protocolized. This typically involved surgeon-preference-based pain management (often PCA-based opioids), variable timing for catheter removal and mobilization, and discharge based on the attending surgeon's general assessment without specific functional criteria.

### Outcome measures

#### Primary outcome

The primary outcome was the change in the Oswestry Disability Index (ODI; version 2.1a) score (11) from baseline to the 2-year follow-up. ODI scores range from 0 to 100, with higher scores indicating greater disability.

#### Secondary outcomes

1. Clinical Scores: Japanese Orthopaedic Association (JOA) score (12) for lumbar disease (0–29 scale, higher is better), and Visual Analog Scale (VAS) for back and leg pain (0–10 scale, 0 is no pain).
2. Quality of Life: The Medical Outcomes Study Short Form-36 (SF-36) (13), yielding a Physical Component Summary (PCS) and a Mental Component Summary (MCS).
3. Radiographic Outcomes: Assessed on standing lateral x-rays at 1 and 2 years. Fusion was defined according to the Bridwell classification (Grade I or II indicating solid fusion). Segmental lordosis and posterior disc height were measured at the index level.
4. Perioperative Metrics: Operative time, intraoperative blood loss, length of hospital stay (LOS), and total opioid consumption (converted to oral morphine equivalents).
5. Complications: All adverse events occurring within 90 days of surgery were recorded, including surgical site infection, dural tear, implant-related issues, deep vein thrombosis (DVT), pulmonary embolism, and medical complications.

### Statistical analysis

The sample size was calculated based on detecting a minimal clinically important difference (MCID) of 5 points in ODI change, with a standard deviation of 12. With a power of 90% and a two-sided alpha of 0.05, a sample size of 170 patients per group was required. To account for a potential 10% dropout rate, we enrolled 191 patients per group. All analyses were performed on an intention-to-treat (ITT) basis. Missing data were handled using multiple imputation. Continuous variables were compared using Student's t-test or Mann–Whitney U test, as appropriate, based on normality testing with the Shapiro–Wilk test. Categorical variables were compared using the Chi-square test or Fisher's exact test. Longitudinal data (ODI, JOA, VAS) were analyzed using a linear mixed-effects model with group, time, and group-by-time interaction as fixed effects and patient as a random effect. A *post-hoc* subgroup analysis was performed based on baseline Body Mass Index (BMI) (non-obese: BMI < 30 kg/m^2^ vs. obese: BMI ≥ 30 kg/m^2^) to evaluate the consistency of the treatment effect on the primary outcome. A *p*-value < 0.05 was considered statistically significant. All analyses were performed using SPSS Statistics v.28.0 (IBM Corp., Armonk, NY, USA).

## Results

### Patient enrollment and characteristics

**Figure 1:**
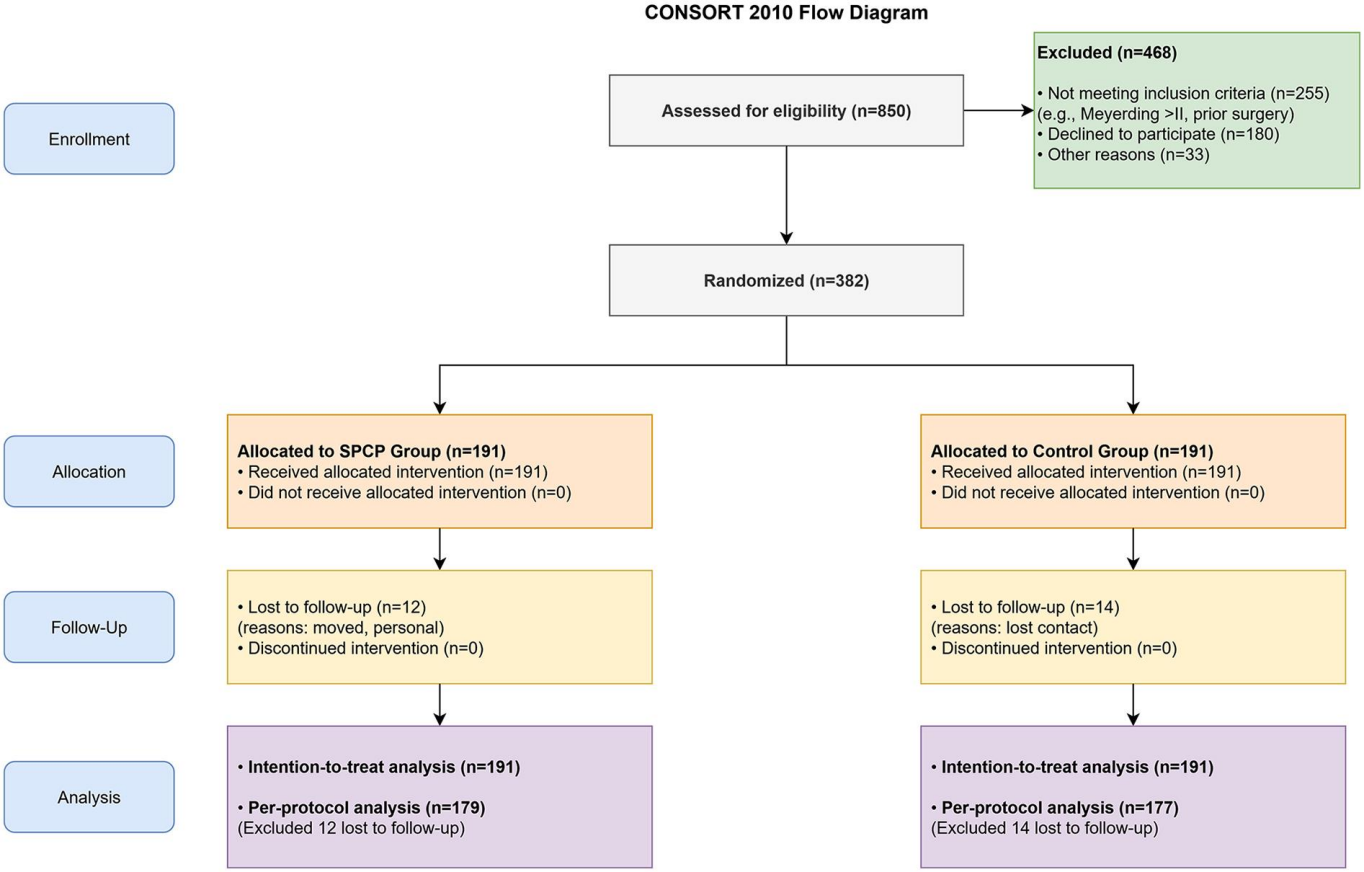
CONSORT flow diagram. Diagram illustrating the flow of participants through each stage of the randomized trial, including enrollment, allocation to the Standardized Perioperative Care Protocol (SPCP) or Control group, follow-up, and analysis. Data are based on the intention-to-treat population.

Of the 850 patients assessed for eligibility, 382 met the criteria and were randomized: 191 to the SPCP group and 191 to the control group. Over the 2-year follow-up period, 12 patients (6.3%) in the SPCP group and 14 patients (7.3%) in the control group were lost to follow-up. The final ITT analysis included all 382 patients (Figure 1). Baseline demographic and clinical characteristics were well-balanced between the two groups, with no statistically significant differences in age, gender, BMI, disease duration, ASA physical status, or preoperative scores (Table 1).

**Table 1:** Baseline demographic and clinical characteristics of patients (intention-to-treat population).

Parameter	SPCP group (*n* = 191)	Control group (*n* = 191)	*P*-value
Age (years), mean ± SD	61.2 ± 9.8	60.8 ± 10.1	0.715
Gender (female), *n* (%)	118 (61.8%)	122 (63.9%)	0.682
BMI (kg/m^2^), mean ± SD	26.5 ± 3.4	26.8 ± 3.6	0.450
Disease duration (months), mean ± SD	28.4 ± 15.1	29.5 ± 16.3	0.589
Meyerding grade, *n* (%)			0.811
Grade I	145 (75.9%)	149 (78.0%)	
Grade II	46 (24.1%)	42 (22.0%)	
Index level, *n* (%)			0.743
L4/5	121 (63.4%)	116 (60.7%)	
L5/S1	58 (30.4%)	65 (34.0%)	
L3/4	12 (6.3%)	10 (5.2%)	
ASA physical status, *n* (%)			0.913
I	51 (26.7%)	49 (25.7%)	
II	122 (63.9%)	125 (65.4%)	
III	18 (9.4%)	17 (8.9%)	
Current smoker, *n* (%)	35 (18.3%)	39 (20.4%)	0.598
Comorbidities, *n* (%)			
Hypertension	61 (31.9%)	65 (34.0%)	0.677
Diabetes mellitus	30 (15.7%)	27 (14.1%)	0.680
Preoperative Scores, mean ± SD			
ODI score	38.5 ± 5.1	38.7 ± 5.3	0.712
JOA score	11.2 ± 2.4	11.0 ± 2.6	0.543
VAS back pain	7.4 ± 1.1	7.5 ± 1.0	0.501
VAS leg pain	6.9 ± 1.3	7.0 ± 1.2	0.588

### Radiographic and clinical outcomes

**Figure 2:**
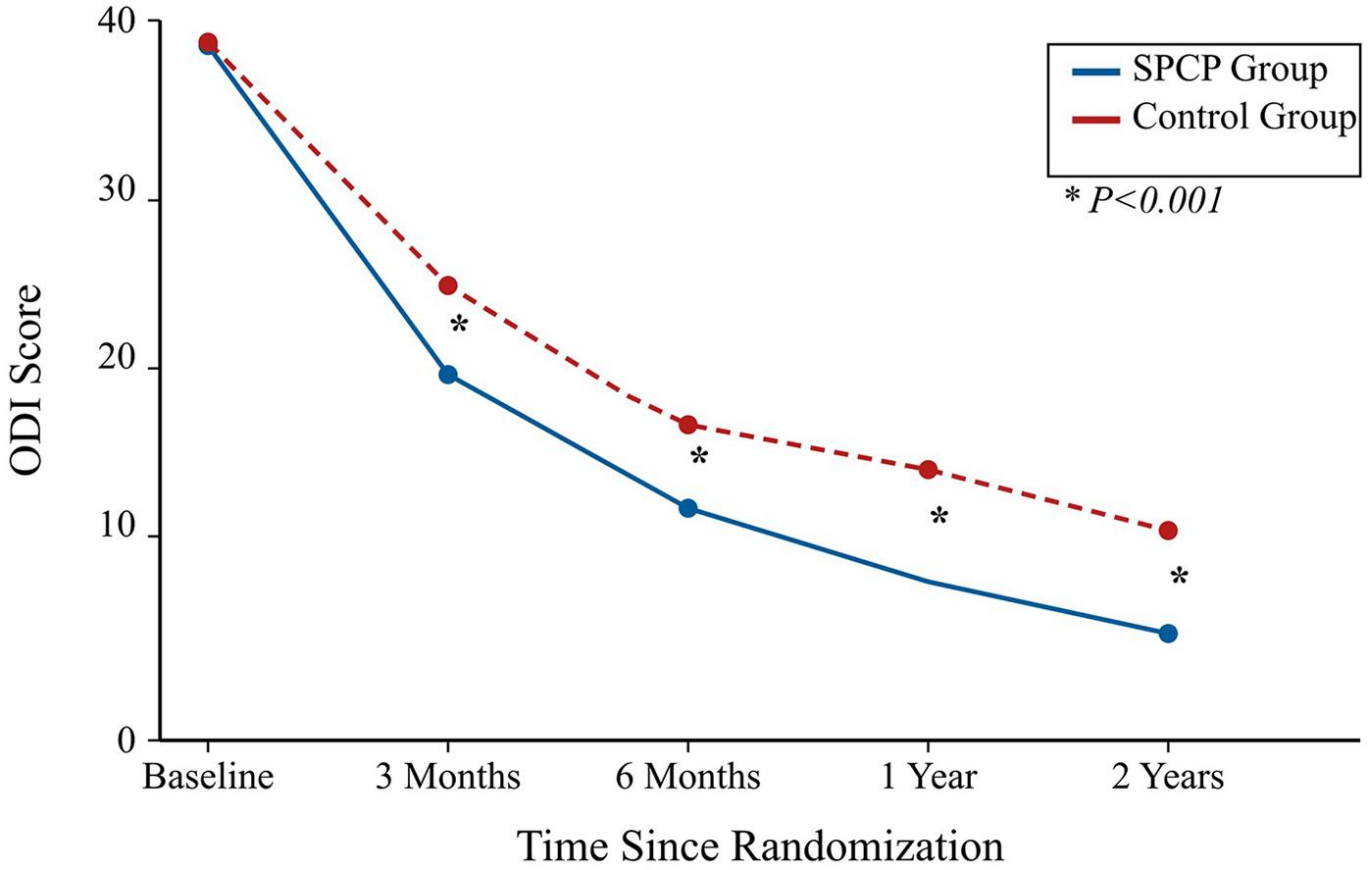
Mean Oswestry Disability Index (ODI) scores over the 2-year follow-up period. Data are presented as mean scores for the SPCP (*n* = 191) and Control (*n* = 191) groups based on the intention-to-treat population. Lower scores indicate less disability. The SPCP group showed significantly greater improvement at all postoperative time points compared to the control group. *P < 0.001 for the group-by-time interaction in the linear mixed-effects model.

**Figure 3:**
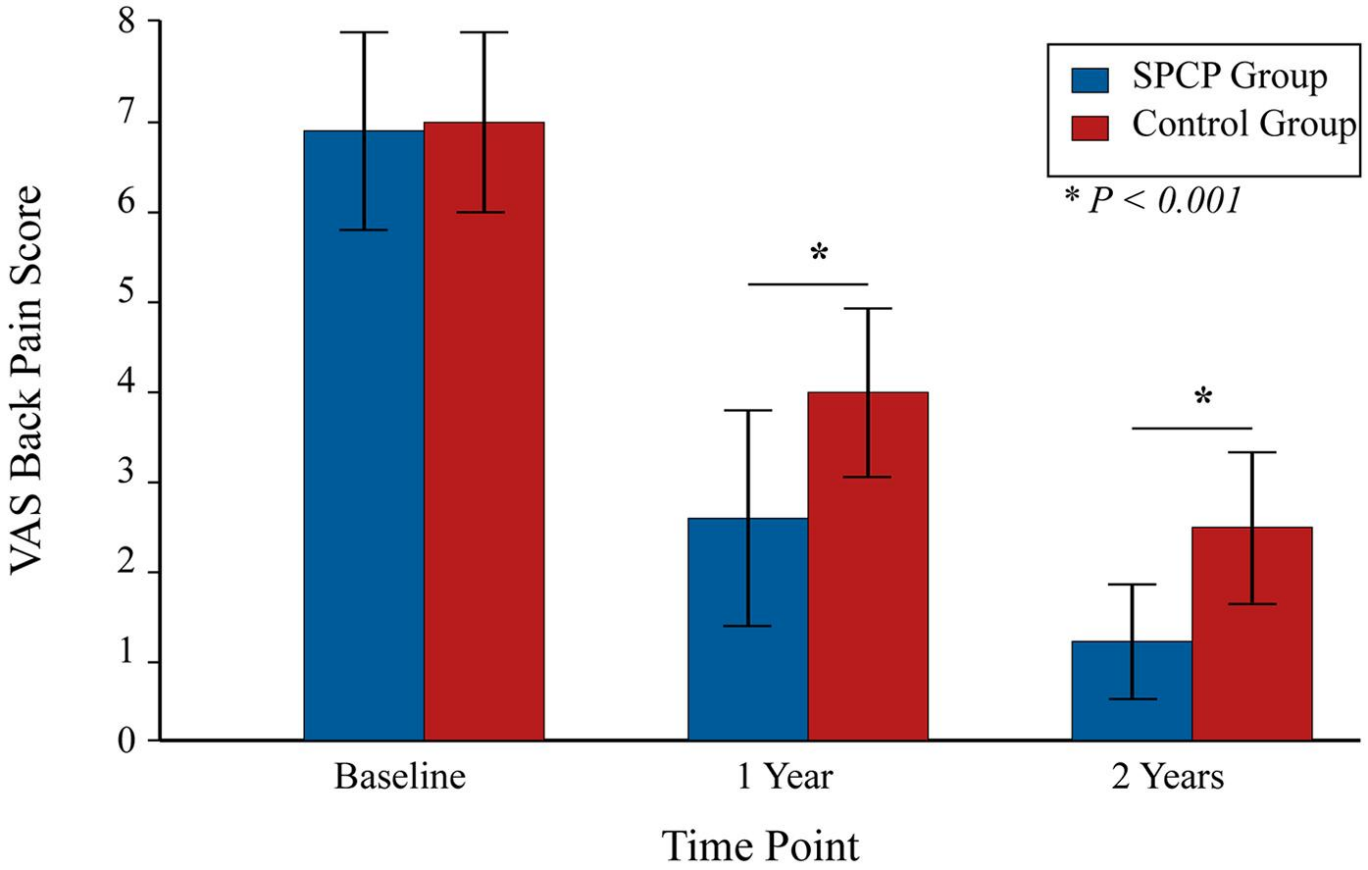
Mean visual analog scale (VAS) scores for back pain. Bars represent the mean VAS score at baseline, 1 year, and 2 years post-randomization for the SPCP and Control groups (*n* = 191 per group). Error bars indicate the standard deviation of the mean. *P < 0.001 for the between-group comparison at the indicated time point using a Student's t-test.

**Figure 4:**
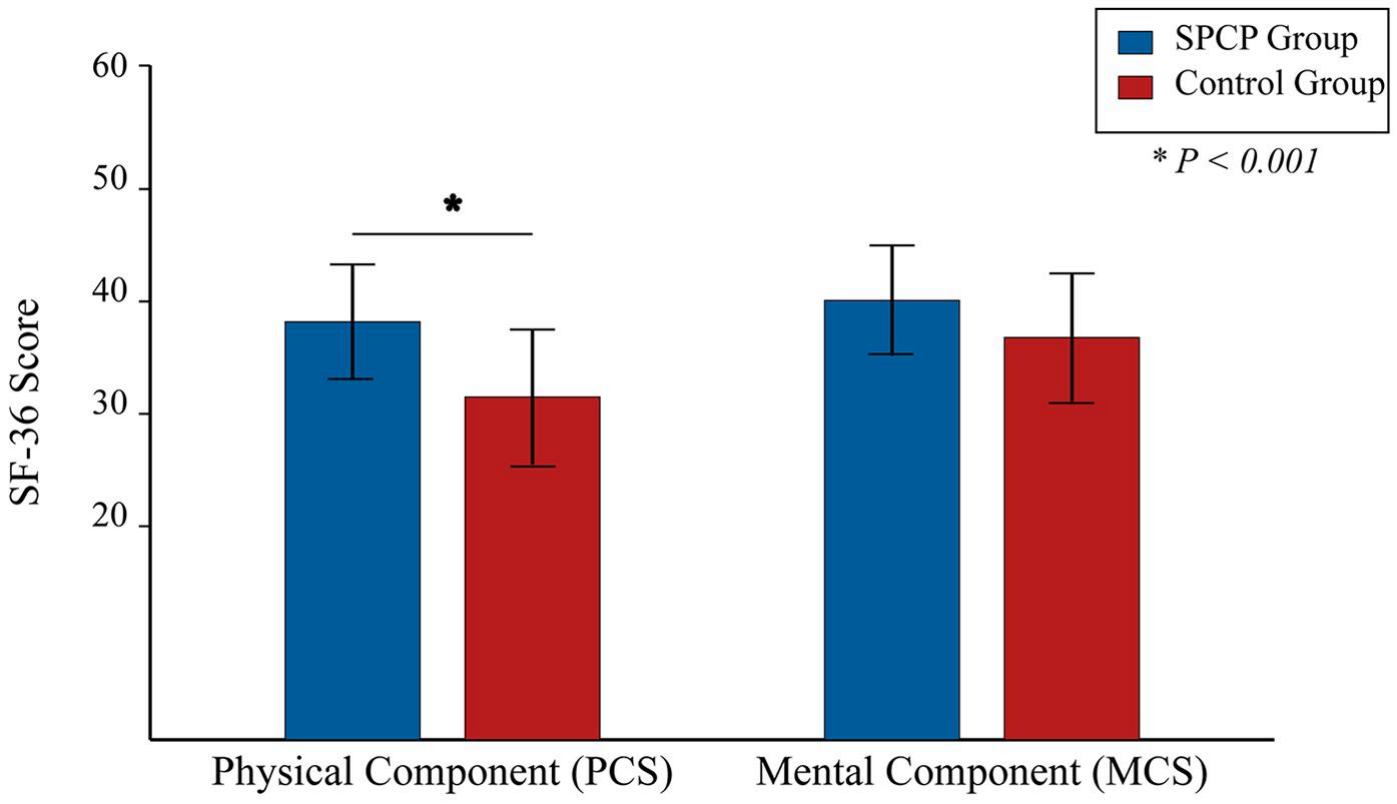
Short form-36 (SF-36) scores at 2-year follow-up. Bars represent the mean scores for the Physical Component Summary (PCS) and Mental Component Summary (MCS) for the SPCP and Control groups (*n* = 191 per group). Higher scores indicate better quality of life. Error bars indicate the standard deviation of the mean. *P < 0.001 for the between-group comparison. The difference for MCS was not statistically significant.

At the 2-year radiographic assessment, the SPCP group demonstrated a significantly higher rate of solid fusion and more favorable sagittal parameters compared to the control group (Table 2). Clinically, both groups showed significant improvements over time. However, the linear mixed-effects model revealed a significant group-by-time interaction favoring the SPCP group for all primary and secondary functional outcomes. At the 2-year endpoint, the SPCP group had achieved a clinically and statistically superior recovery in terms of disability (ODI), function (JOA), pain (VAS), and quality of life (SF-36) (Table 3; Figures 2–4).

**Table 2:** Radiographic outcomes at 2-year follow-up (per-protocol population).

Parameter	SPCP group (*n* = 179)	Control group (*n* = 177)	*P*-value
Fusion rate (Bridwell I or II), *n* (%)	169 (94.4%)	157 (88.7%)	**0**.**018**
Change in segmental lordosis (degrees), mean ± SD	+4.5 ± 2.1	+3.1 ± 2.5	**<0**.**001**
Change in posterior disc height (mm), mean ± SD	+3.8 ± 1.5	+2.9 ± 1.8	**<0**.**001**
Cage subsidence (>2 mm), *n* (%)	9 (5.0%)	20 (11.3%)	**0**.**035**

Bold values indicate statistical significance (*P* < 0.05).

**Table 3:** Comparison of clinical outcome scores over time (ITT population).

Indicator	Time point	SPCP group (*n* = 191)	Control group (*n* = 191)	Mean difference (95% CI)	*P*-value
ODI score	Baseline	38.5 ± 5.1	38.7 ± 5.3	−0.2 (−1.5 to 1.1)	0.712
	3 Months	18.2 ± 4.9	23.5 ± 5.8	−5.3 (−6.5 to −4.1)	**<0**.**001**
	6 Months	13.1 ± 4.5	17.9 ± 5.2	−4.8 (−6.0 to −3.6)	**<0**.**001**
	1 Year	10.3 ± 4.2	15.4 ± 5.4	−5.1 (−6.3 to −3.9)	**<0**.**001**
	2 Years	8.5 ± 4.1	13.2 ± 5.5	−4.7 (−5.9 to −3.5)	**<0**.**001**
JOA score	Baseline	11.2 ± 2.4	11.0 ± 2.6	0.2 (−0.5 to 0.9)	0.543
	3 Months	22.5 ± 2.8	18.9 ± 3.1	3.6 (2.9 to 4.3)	**<0**.**001**
	6 Months	25.1 ± 2.5	21.2 ± 3.0	3.9 (3.2 to 4.6)	**<0**.**001**
	1 Year	26.8 ± 2.1	22.5 ± 2.9	4.3 (3.6 to 5.0)	**<0**.**001**
	2 Years	27.5 ± 2.0	23.1 ± 2.8	4.4 (3.7 to 5.1)	**<0**.**001**

Bold values indicate statistical significance (*P* < 0.05).

### Perioperative outcomes and complications

Operative time and intraoperative blood loss were comparable between groups. However, the mean length of hospital stay was significantly shorter for the SPCP group (7.5 ± 2.1 days vs. 9.8 ± 2.5 days, *P* < 0.001). As detailed in Table 4, total postoperative opioid consumption was also significantly lower in the SPCP group (mean 85 ± 35 mg oral morphine equivalents) compared to the control group (mean 142 ± 51 mg oral morphine equivalents, *P* < 0.001). The SPCP group also had a significantly lower overall 90-day complication rate (8.4% vs. 19.4%, *P* = 0.002), driven primarily by lower rates of ileus and urinary retention (Table 4).

**Table 4:** Comparison of perioperative outcomes and 90-Day complications (ITT population).

Parameter	SPCP group (*n* = 191)	Control group (*n* = 191)	*P*-value
Operative time (min), mean ± SD	175.4 ± 35.2	179.1 ± 38.6	0.381
Blood loss (mL), mean ± SD	355 ± 150	370 ± 165	0.429
Length of hospital stay (days), mean ± SD	7.5 ± 2.1	9.8 ± 2.5	**<0**.**001**
Complications, *n* (%)			
Superficial surgical site infection	3 (1.6%)	5 (2.6%)	0.498
Dural tear (incidental)	4 (2.1%)	6 (3.1%)	0.545
Deep vein thrombosis (DVT)	2 (1.0%)	4 (2.1%)	0.449
Pulmonary embolism	0 (0%)	1 (0.5%)	0.499
Ileus	3 (1.6%)	12 (6.3%)	**0**.**017**
Urinary retention (requiring recatheterization)	4 (2.1%)	16 (8.4%)	**0**.**006**
Reoperation (within 90 days)	1 (0.5%)	3 (1.6%)	0.623
Total complications (any)	16 (8.4%)	37 (19.4%)	**0**.**002**
Total opioid consumption (oral morphine equivalents, mg), mean ± SD	85 ± 35	142 ± 51	**<0**.**001**

Bold values indicate statistical significance (*P* < 0.05).

### Subgroup analysis

The *post-hoc* subgroup analysis stratified by baseline BMI showed that the SPCP was associated with a greater improvement in ODI scores at 2 years compared to conventional care in both non-obese (BMI < 30 kg/m^2^) and obese (BMI ≥ 30 kg/m^2^) patients (Supplementary Table S1). In the non-obese subgroup, the mean difference in ODI change was −4.9 (95% CI: −6.4 to −3.4; *P* < 0.001), while in the obese subgroup, the mean difference was −4.2 (95% CI: −5.9 to −2.5; *P* < 0.001). There was no significant interaction between the treatment effect and BMI category (*P* for interaction = 0.481), suggesting that the benefit of the SPCP was consistent across different BMI strata.

## Discussion

This randomized controlled trial demonstrates that the implementation of a comprehensive Standardized Perioperative Care Protocol (SPCP) leads to significantly improved outcomes for patients undergoing TLIF for degenerative spondylolisthesis. Compared to conventional care, the SPCP group achieved superior functional recovery, better radiographic fusion, higher quality of life, a shorter hospital stay, and a lower rate of postoperative complications. These findings provide robust evidence supporting the value of protocol-driven, multidisciplinary care in the context of complex spine surgery.

The primary outcome, a greater improvement in the ODI score, is both statistically and clinically significant. The mean difference of 4.7 points at 2 years exceeds the established minimal clinically important difference, suggesting a tangible benefit for patients in the SPCP group (14). This enhanced functional recovery is likely multifactorial. Preoperative education may have better aligned patient expectations and improved adherence to postoperative instructions. The structured, goal-directed rehabilitation, starting on POD 1, likely prevented deconditioning, reduced pain through early mobilization, and fostered patient confidence. This is consistent with ERAS principles, where early mobilization is a cornerstone for reducing pulmonary complications, ileus, and VTE risk (7, 15). Indeed, our study showed significantly lower rates of ileus and urinary retention in the SPCP group, complications directly linked to immobility and opioid use.

A novel finding of our study is the significant improvement in radiographic outcomes. The SPCP group had a higher fusion rate and better maintenance of sagittal parameters at 2 years. While the link between clinical and radiographic outcomes can be tenuous, achieving a solid fusion is a fundamental goal of TLIF. The mechanism for this finding is speculative but may relate to the holistic nature of the protocol. Specifically, elements of the SPCP may create a more favorable biological environment for bone healing. Optimized preoperative nutrition can improve serum albumin levels, a key factor for wound healing and bone formation (16). The opioid-sparing analgesia approach reduces systemic inflammation, which can otherwise inhibit osteoblast activity (17). Furthermore, early and structured mobilization may enhance local blood flow to the fusion site, delivering essential nutrients and growth factors (18, 19). A recent systematic review has corroborated that such multimodal perioperative strategies positively influence the molecular signaling pathways crucial for successful spinal arthrodesis (20). Furthermore, the lower incidence of cage subsidence may contribute to better long-term preservation of foraminal height and sagittal alignment, which is correlated with better clinical outcomes (21).

The 2.3-day reduction in length of hospital stay is a critical finding from a healthcare economics perspective. This reduction was achieved not by premature discharge but by facilitating a faster return to functional independence. This efficiency gain, coupled with a lower complication rate, suggests that SPCPs can reduce the overall cost of care while simultaneously improving its quality, a key tenet of value-based healthcare (22).

Our results build upon previous work on ERAS in spine surgery. While many studies have shown benefits in LOS and opioid consumption (7, 23, 24), few have been large-scale RCTs with long-term follow-up and a comprehensive assessment of functional and radiographic outcomes (25, 26). Our 2-year follow-up provides crucial insights into the sustainability of the observed benefits, demonstrating that the advantages of the SPCP are not transient but are maintained long after discharge.

This study has several limitations. First, as a single-center trial, the results may be influenced by local expertise and resources, potentially limiting generalizability. A multicenter trial would be necessary to validate these findings across different healthcare systems. Second, the inability to blind surgeons and patients to the intervention introduces a potential for performance and placebo effects. However, the use of blinded outcome assessors and objective radiographic measures mitigates this bias. Third, we did not perform a formal cost-effectiveness analysis, which would be a valuable future investigation to quantify the economic benefits of the SPCP. Fourth, the trial was registered retrospectively, which is a deviation from the ideal prospective registration recommended by the CONSORT statement. Fifth, our assessment of fusion was based on dynamic plain radiographs rather than computed tomography (CT) scans. While the Bridwell classification on x-ray is a validated and widely used clinical standard, CT remains the gold standard for definitively confirming bony fusion (27, 28). This methodological choice, made to reduce radiation exposure and align with routine follow-up protocols, represents a limitation, and the superior fusion rate in the SPCP group should be interpreted with this in mind; further investigation with advanced imaging could validate this finding more robustly. Finally, while we had a low overall attrition rate, the loss to follow-up could still introduce bias.

## Conclusion

In conclusion, a comprehensive Standardized Perioperative Care Protocol (SPCP) significantly improves long-term functional recovery, radiographic fusion rates, and quality of life while reducing length of stay and postoperative complications in patients undergoing TLIF for degenerative spondylolisthesis. The integration of preoperative optimization, standardized intraoperative techniques, and goal-directed postoperative rehabilitation should be considered a new standard of care to enhance the value and efficacy of spine fusion surgery.

## Data Availability

The original contributions presented in the study are included in the article/Supplementary Material, further inquiries can be directed to the corresponding author.
