# Supplementary material for: Impact of a standardized perioperative care protocol on functional and radiographic outcomes following transforaminal lumbar interbody fusion for degenerative spondylolisthesis: a 2-year randomized controlled trial
Source: Front Surg. 2025 Dec 18;12:1679851. doi: 10.3389/fsurg.2025.1679851 (PMC12756368; doi:10.3389/fsurg.2025.1679851)
Supplement: Supplementary file 1 [file Table1.docx]

**Table S1. Subgroup analysis of the change in ODI score from baseline to 2 years, stratified by Body Mass Index (BMI).**

| **BMI Subgroup** | **Parameter** | **SPCP Group** | **Control Group** | **P-value** |
| --- | --- | --- | --- | --- |
| **Non-obese (BMI < 30 kg/m²)** | n | 138 | 135 |  |
|  | Change in ODI, mean ± SD | -30.8 ± 5.9 | -25.9 ± 6.2 | <0.001 |
| **Obese (BMI ≥ 30 kg/m²)** | n | 53 | 56 |  |
|  | Change in ODI, mean ± SD | -28.1 ± 6.4 | -23.9 ± 6.8 | <0.001 |
| **P for interaction between treatment and BMI subgroup** | | | | 0.481 |
